# Children’s Elaboration of Forensically Relevant Information in Response to Invitations: A National Study of Investigative Interviews With Preschool-Aged Abuse Victims

**DOI:** 10.1177/10775595251328933

**Published:** 2025-04-11

**Authors:** Miriam S. Johnson, Rolf Magnus Grung, Ragnhild K. Røed, Are Hugo Pripp, Gunn Astrid Baugerud

**Affiliations:** 1 Faculty of Health Sciences, Oslo Metropolitan University, Oslo, Norway; 2 Department of Social Work, Child Welfare and Social Policy, Faculty of Social Sciences, Oslo Metropolitan University, Oslo, Norway

**Keywords:** investigative interviews, child victims, invitations, children’s elaboration

## Abstract

This field study investigated the use and efficacy of main and cued invitations in eliciting forensically relevant information from a national sample of forensic interviews conducted with preschool-aged (2- to 6-year-old) alleged victims of abuse. Among 1065 invitations posed by the interviewers, 43 (4%) were classified as main invitations, while 1022 (96%) were identified as cued invitations. Both subtypes of invitations were equally effective in eliciting event-specific, forensically relevant information from the children. Nearly 70% of main invitations yielded forensically relevant information, compared to 83% for cued invitations. Interviewers typically presented only one invitation before resorting to other prompts, predominantly directive, option-posing, and suggestive questions. The effectiveness of invitations increased significantly when posed later in the interviews, suggesting a decline in children’s productive responses as the interview progressed. This study highlights potential barriers to the effective use of invitations and discusses implications for developing tailored training programs for interviewers working with preschool-aged alleged victims.

## Introduction

Preschool-aged children represent a significant proportion of sexual abuse victims, with research estimating that 25%–35% of these victims are under the age of six (Brilleslijper-Kater et al., 2004). Nevertheless, many instances of child abuse go unreported, and official figures likely capture only a fraction of actual cases, as approximately 30% of cases are never brought to the attention of authorities (Yüksel & Koçtürk, 2020). This underreporting is particularly concerning given the increased vulnerability of preschool-aged children, who are more dependent on their caregivers and have greater difficulty verbalizing their experiences of abuse (Lamb et al., 2003; London et al., 2005). Moreover, preschoolers are generally more reluctant to disclose abuse in formal forensic interviews compared to older children (Azzopardi et al., 2019; London et al., 2005). Given the multiple barriers that can lead to abuse being overlooked or unreported, it is crucial for interviewers to employ effective, developmentally appropriate techniques that facilitate spontaneous, accurate, and detailed information from young, alleged victims of abuse (Hershkowitz et al., 2005; Korkman et al., 2024; Leach et al., 2016; Powell, 2013; Powell & Snow, 2007; Røed et al., 2025). A key factor in eliciting such narrative accounts from preschoolers is largely dependent on the types of questions interviewers ask in forensic interviews (Cyr & Lamb, 2009; Korkman et al., 2024; Lamb et al., 2008; Lamb & Fauchier, 2001; Peterson et al., 1999; Powell, 2013; Sternberg et al., 1996; Talwar et al., 2024). Extensive research has consistently demonstrated that open-ended prompts—that encourage free and detailed, narrative accounts without restricting or leading the interviewee—are the most effective for eliciting accurate and detailed information compared to information retrieved in response to directive, option-posing, or suggestive prompts (Cederborg et al., 2000; Cyr & Lamb, 2009; Danby & Sharman, 2023; Gagnon & Cyr, 2017; Hershkowitz, 2001; Lamb et al., 2008). Children’s responses to open-ended prompts have been found to be three to five times more informative than those elicited by more focused prompts (Lamb et al., 1996, 2003, 2008; Sternberg et al., 2001) In forensic interviews, the use of open-ended prompts has also been shown to facilitate the retrieval of forensically relevant information about investigated incidents (Cyr & Lamb, 2009; Danby & Sharman, 2023; Gagnon & Cyr, 2017; Hershkowitz, 2001; Lamb et al., 2007b; Orbach et al., 2000; Sternberg et al., 2001). The robustness of information elicited through open-ended prompts is notable, as it tends to remain accurate over time, in contrast to the decline in accuracy often seen with directive, option-posing, and suggestive questions (Brown et al., 2013; Lamb et al., 2003, 2007b; Leichtman & Ceci, 1995; Poole & Lamb, 1998). Additionally, researchers have also reported low rates of fabrication errors and infrequent instances of contradictory details in children’s responses to open-ended prompts (Brubacher et al., 2019; Lamb & Fauchier, 2001). In addition to the positive effects of open-ended questions on children’s responses, research has shown that their use is associated with interviewer competencies essential for the overall quality of the interview. For example, interviewers’ use of open-ended prompts is often supported by other best-practice interviewing behaviors, such as employing developmentally appropriate techniques, using age-appropriate language, and minimizing the risk of suggestive questioning (Brown & Lamb, 2015; La Rooy et al., 2015). Consequently, these robust findings on the superiority of open-ended prompts have informed professional guidelines, which recommend that interviewers predominantly use open-ended prompts to elicit children’s free narrative accounts in forensic interviews (Brown & Lamb, 2015; Bull, 2011; Lamb, 2016; Lamb et al., 2008; Orbach et al., 2000; Powell, 2013).

While the superiority of open-ended prompts is well established in school-aged children in forensic interviews, their effectiveness in eliciting detailed and accurate responses from preschool-aged children has been a topic of considerable discussion in the literature. Researchers have emphasized that preschoolers’ responses to open-ended prompts often tend to be brief and incomplete (Hewitt, 1999). Factors such as shorter attention spans, less sophisticated language skills, and underdeveloped memory retrieval strategies in younger children can affect their ability to provide rich, coherent, and detailed accounts compared to older children (Lamb et al., 2008; Saywitz et al., 2018). Preschoolers may also struggle with understanding concepts such as time and event sequencing, which can impact the overall coherence and level of detail in their narratives (Poole & Bruck, 2012; Saywitz et al., 2018). However, while age and developmental immaturity can limit preschoolers’ capacity to report detailed and extensive information about remembered events, their accuracy for recalled events is often similar to that of older children (Gagnon & Cyr, 2017; Lamb et al., 2008). Although young children’s responses may not match those of older children in terms of details and length, several studies have demonstrated that children as young as 3–4 years old can provide forensically relevant information when prompted with open-ended questions (Cederborg et al., 2000; Gagnon & Cyr, 2017; Hershkowitz et al., 2012; Lamb et al., 2003; Sternberg et al., 2001). Importantly, preschoolers’ free-recall reports have been found to be as accurate as those of older children (Lamb et al., 2003). In many cases, preschoolers can yield as much information in response to open-ended questions as their older counterparts, including forensically relevant information (Cederborg et al., 2000; Hershkowitz et al., 2012; Sternberg et al., 2001). For instance, Lamb et al. (2003) showed that children as young as four years of age can provide substantial amounts of forensically important information about alleged abuse in response to free-recall prompts. Although older children reported more details in total and in their average responses to invitations than the younger children, the proportion of details elicited using free-recall prompts did not increase with age. On average, invitations also elicited more forensically relevant details than other types of questions at all ages. Despite older children providing progressively longer and more detailed responses with age, children as young as 4 were capable of providing at least half of the information through open-ended invitations (Lamb et al., 2003). Similar findings regarding younger children’s capacity have been reported in other studies (e.g., Lamb et al., 1996, 2007b; Orbach et al., 2000; Sternberg et al., 2010). Despite these findings, other studies indicate that preschoolers generally require more support in recalling specific details. As a result, specific questions are believed to be more effective for eliciting information from young children. For instance, a large-scale study by Hershkowitz et al. (2012) examined age differences in 299 preschoolers’ responses to investigative interviewers’ questions. The findings showed that children aged 3–4 tended to respond slightly more informatively to specific, directive prompts compared to open-ended prompts. In contrast, children aged 5 and older were more informative in response to open-ended prompts. These findings suggest that while 3-year-olds can provide information about experienced events when recall processes are activated, the ability to provide narrative responses to open-ended prompts becomes more reliable as children develop. Similar findings were reported in a study by Gagnon and Cyr (2017), which examined the association between subtypes of questions (e.g., open-ended directive and closed-ended) used in 55 forensic interviews with preschoolers aged 3–5 years who disclosed an episode of sexual abuse. In this study, children provided more details in response to invitations compared to other question types. However, responses to cued open-ended questions – those that prompted the child to elaborate on a previously mentioned detail – elicited more specific details about the incident compared to general invitations. Similar to the Hershkowitz et al. (2012) study, these findings suggest that using open-ended questions with cues already mentioned in the child’s testimony helps obtain a more detailed account during an investigative interview with preschoolers (Gagnon & Cyr, 2017).

In summary, the existing research on younger children’s retrieval capacity in response to open-ended prompts presents two key findings. Some evidence challenges prior assumptions that young children are less capable of providing informative responses and may not offer substantial information without more focused prompts. On the other hand, other studies suggest that preschoolers may require additional support to maintain focus and recall specific details in forensic interviews. These inconsistent findings highlight the need for further investigation into the efficacy of different subtypes of open-ended prompts when interviewing preschoolers. To address these issues, several key factors should be comprehensively explored in future research. First, most field studies on the effectiveness of open-ended prompts in forensic interviews have focused on school-aged and adolescent children (Cyr & Lamb, 2009; Danby & Sharman, 2023; Hershkowitz, 2001; Orbach et al., 2000; Sternberg et al., 2001). While many studies include a broad age range, encompassing preschool-aged children, school-aged children, and adolescents, the proportion of interviews with preschoolers remains low. This limited focus restricts the ability to thoroughly examine preschoolers’ specific response patterns. As a result, field studies focusing exclusively on samples of preschool-aged children are scarce in the current literature, leaving a significant gap in understanding the efficacy and mechanisms of open-ended prompts for this group. Secondly, over the past two decades, research across various countries—including Canada (Luther et al., 2015; Roberst & Cameron, 2015), France (Verkampt et al., 2021), the Netherlands (Otgaar et al., 2019), Israel (Lamb et al., 1996; Orbach et al., 2000), Korea (Yi et al., 2015), Sweden (Cederborg et al., 2000), Norway (Baugerud et al., 2020, 2023; Johnson et al., 2015), Finland (Korkman et al., 2008), and Australia (Powell & Hughes-Scholes, 2009)—has consistently shown that open-ended prompts are seldom used in investigative interviews with children at all ages. Despite the well-documented efficacy of open-ended prompts in eliciting detailed and accurate information, interviewers often rely on option-posing or suggestive prompts (Cederborg et al., 2000; Korkman et al., 2008). The underuse of open-ended prompts limits opportunities for systematic study of their impact in large-scale, real-world settings, particularly with preschool-aged children. Therefore, large-scale studies examining the efficacy of these prompts are needed.

Another key factor that warrants further examination is the investigation of different subtypes of open-ended prompts within large samples of preschoolers. To date, few studies have closely examined preschoolers’ response patterns to various subtypes of open-ended prompts in large samples of investigative interviews. Open-ended prompts vary in characteristics and scope in several ways (Lamb et al., 2003; Orbach & Lamb, 2000). Specifically, these prompts differ in their level of specificity. General or main invitations - also referred to as initial broad open-ended invitations - encourage narrative responses by asking the child to recount an event broadly in their own words, with minimal direction or prompting beyond the initial request (e.g., “Tell me what happened” or “Tell me everything you can remember. Start at the beginning”) (Lamb et al., 2003; Orbach & Lamb, 2000; Wright & Powell, 2006). Main invitations are designed to elicit free recall, allowing children to remember and describe events without being led to specific details. In contrast, cued invitations have distinct characteristics that set them apart from main invitations. These prompts are more specific and build on the child’s previous responses or disclosed information, encouraging them to elaborate on particular aspects of an event. Cued invitations can help clarify or expand on elements of a narrative that may be vague or incomplete. For example, a cue might ask for more information about a person, action, or setting previously mentioned by the child, prompting further free-recall elaboration (Lamb et al., 2003, 2007a). Cued invitations typically follow an initial main invitation and serve as follow-up prompts to deepen the child’s narrative and uncover additional information about the event in question. This variability in characteristics and scope of open-ended prompts can significantly affect the quality and quantity of information elicited from preschoolers during forensic interviews. Additionally, there is a lack of understanding regarding preschoolers’ response patterns to main versus cued invitations. Given the developmental differences among preschoolers of varying ages, it is plausible that younger children may find it more challenging to respond informatively to general invitations compared to their older counterparts (Hershkowitz et al., 2012). Therefore, further research is needed to examine the effectiveness of different subtypes of invitations and how young children respond to them (Lamb et al., 2008; Poole & Bruck, 2012). In summary, the existing body of research underscores the need for studies that explore patterns in interviewers’ use of invitations in real-life cases. Specifically, there is a need for large-scale studies focusing on the efficacy of different subtypes of invitations with preschool-aged children.

The present study aims to address these gaps in the literature by investigating interviewers’ use and efficacy of two subtypes of invitations (i.e., main and cued invitations) and children’s narrative elaboration of forensically relevant information in response to these invitations. The sample consists of 256 investigative interviews with preschool-aged children, ranging from 2 to 6 years old. The interviews stem from severe cases that were part of formal criminal investigations into alleged physical and sexual abuse against preschool-aged children, all of which proceeded to prosecution. The study has several key objectives. First, we aim to explore the overall frequency and distribution of main and cued invitations during the substantive phase of the interviews. Following the consensus recommendation that invitations should be introduced early in the substantive phase to elicit free-recall memory of the incidents under investigation, we examined the distribution and timing of the first main and cued invitations during this phase. Specifically, we analyzed in which turn (i.e., pairs of questions and responses) these invitations were posed throughout the interviews and how their timing was associated with children’s elaboration of forensically relevant information. Consistent with previous research indicating that interviewers typically present only one invitation before shifting to other question types (Korkman et al., 2008; Wolfman et al., 2016), we examined whether interviewers continued to present additional invitations following the first main invitation. Additionally, we further examined children’s responses to main versus cued invitations, with a particular emphasis on the retrieval of event-specific, forensically relevant information that described participants, settings, actions, and conversations relevant for the investigated incident. Based on the assumption that younger children may have difficulty responding informatively to main invitations (Guadagno et al., 2006, 2013; Wright & Powell, 2006), we examined whether elaboration of forensically relevant information varied among preschoolers in the lower (2–4 years old) and upper (5–6 years old) age groups. Lastly, we examined the distribution of interviewers’ follow-up prompts after the children’s elaboration of forensically relevant information in response to main and cued invitations, as well as children’s elaboration in response to these follow-up prompts.

## Method

### Sample

We examined transcripts from a nationally representative cohort of 256 forensic interviews with preschool-aged children (71%, *n* = 183 female), aged 2–6 years (*M* = 4.9, *SD* = 9.56, age range = 34–79 months). The interviews were randomly selected from a pool of investigative interviews with preschool-aged children (*n* = 550) conducted within 12 police regions in Norway between 2015 and 2017. These interviews were part of formal criminal investigations into suspected cases of physical and sexual abuse against children that proceeded to prosecution. The sample was drawn from all police regions in the country, ensuring geographic representation of alleged child abuse cases from both urban and rural areas, and from diverse socioeconomic backgrounds. The interviewees were alleged victims of physical abuse (*n* = 155, 60.5%) or sexual abuse (*n* = 101, 39.5%). The nature of the alleged abuse varied in severity, with reported incidents including spanking, beating, exposure, fondling of the perpetrator’s genitals, and penetration. Most cases (*n* = 238, 93%) involved alleged intra-familial abuse. The age distribution was as follows: 2- and 3-year-olds (*n* = 34, age range = 34–47 months), 4-year-olds (*n* = 95, age range = 48–59 months), 5-year-olds (*n* = 84, age range = 60–71 months), and 6-year-olds (*n* = 43, age range = 72–79 months). It was confirmed in the transcripts that none of the children in the sample were attending school at the time of the interview, thus classifying them as preschool-aged children. For analytical purposes, we categorized the children into two age groups: 2- to 4-year-olds (*n* = 129) and 5- to 6-year-olds (*n* = 127). This research is part of a national study funded by the Ministry of Justice and Public Security, which aims to investigate the quality of forensic interviews in a national sample of preschool-aged children.

Ethics approval was obtained from the State Attorney, the National Police Directorate, the Data Inspectorate, and the University’s Data Protection Office.

### Interviewers and Interview Method

The interviews were conducted by police investigators from 12 police regions, with 79.3% (*n* = 203) being female and 20.7% (*n* = 53) male. All investigators had undergone identical training in the national child witness interviewing model known as the Sequential Interview Model (SI-model). Specific details regarding the interviewers’ individual levels of experience were not available. The SI-model was developed and implemented nationwide to guide investigative interviews with vulnerable witnesses, including preschool-aged children and individuals with disabilities (Langballe & Davik, 2017). The SI-model comprises three key phases. The initial pre-substantive phase focuses on building rapport with the child, creating a supportive environment, and familiarizing the child with the interview structure. This phase includes setting ground rules and practicing them with the child. Following this, the substantive phase explores the alleged event, typically over multiple sessions with breaks in between, using open-ended questions to encourage free narrative recall. Directive questions are employed to clarify or expand on the child’s account, while suggestive and closed questions are intentionally avoided). The closing phase concludes the interview and addresses the child’s well-being. The SI-model emphasizes the use of open-ended questions to promote free narrative recall, with directive questions used to clarify or elaborate on the child’s statements. Suggestive and closed questions are deliberately avoided to ensure the integrity of the child’s testimony (Langballe & Davik, 2017).

### Procedure

#### Coding

All personal information was removed from the transcripts during verbatim coding to ensure anonymity and confidentiality during data collection. Main and cued invitations, children’s responses to these invitations, and interviewers’ follow-up questions were extracted from a larger data file coded for question types and children’s responses in the substantive phase of the interviews, which focused specifically on the investigated incident(s) (see La Rooy & Lamb, 2011; Szojka & Lyon, 2023, for a similar procedure). Prompts that focused on questions not related to the investigated incident(s) were not included in the analysis.

Interviewers’ prompts were coded in accordance with the guidelines outlined in the National Institute of Child Health and Human Development (NICHD) investigative interview coding scheme (Hershkowitz et al., 2012; Lamb et al., 2003; 2007a; La Rooy et al., 2015). This coding procedure distinguished between main and cued invitations. Main invitations are open-ended prompts that encourage the child to recall information about the incidents, typically in the form of a question, statement, or imperative (e.g., “Tell me everything that happened.” or “Tell me everything that happened from the beginning to the end as best you can remember”). Cued invitations, in contrast, build on specific details mentioned by the child, using those details as cues to elicit further free recall of forensically relevant information related to the alleged incidents (e.g., “You mentioned (event, action, object), tell me more about that” or “Then what happened?”). Children’s responses to invitations were categorized as either on-track or off-track responses (see Lamb et al., 2007b; Szojka & Lyon, 2023, for a similar procedure). On-track responses included affirmative responses that specifically addressed the issue raised by the interviewer and contained the requested information. These responses were further divided into two subcategories: (1) responses that verbally confirmed the investigated incidents without providing additional information or details and (2) responses that verbally confirmed the investigated incidents while also identifying or describing forensically relevant information, including event-specific details about participants, objects, actions, events, and locations. This categorization was inspired by the narrative elaboration procedure (Saywitz et al., 2018), which incorporates visual cues representing four retrieval categories: participants, settings, actions, and conversations. Within the category of on-track responses, the raters also distinguished between responses that provided new, forensically relevant information and those that did not. Off-track responses included verbal responses that did not provide the requested information or did not address the issue raised by the interviewers. These included expressions of lack of memory, knowledge, or understanding (i.e., “I don’t know.” “I don’t remember.” “I don’t understand.”), refusals to answer (“I don’t want to answer that question.”), verbal responses providing substantive information that was not requested, denial (e.g., “Nothing happened), corrective responses (e.g., “No, I already told you, he did not hit me.”), and no response at all. Additionally, responses that provided substantive but irrelevant information were also categorized as off-track.

Interviewers’ follow-up prompts after children’s responses to invitations were coded as either directive, option-posing, or suggestive prompts (Lamb et al., 1996, 2007a). Directive questions focused on obtaining additional information previously mentioned by the child, typically using wh-questions (e.g., ‘When did it happen?’ or ‘What color was his car?’ after the child mentioned a car). Option-posing questions directed the child’s attention to aspects not previously mentioned by them, often requiring confirmation or selecting from specific options provided by the interviewer. These included yes/no prompts (e.g., “Did he hit you?”) or forced-choice prompts, where the interviewer presents pre-defined responses for the child to choose from (e.g., “Did he touch you over or under your clothes?”). Suggestive questions, on the other hand, implied a specific response or introduced details not previously mentioned by the child (e.g., “He was doing something bad to you that you did not like, wasn’t he?’ or ‘Did he say anything when he touched you?’ when the child had not mentioned being touched).

Facilitators, which include non-suggestive prompts, were also coded. These support or rephrase the child’s ongoing response in a neutral manner (e.g., “OK,” “Yes,” “Uh-huh,” or “So he hit you” immediately following the child’s statement, “and then he hit me”) (Lamb et al., 2007a). Given that this study is a field investigation involving children recounting their experiences without corroborating evidence to validate the information provided, assessing the accuracy of the children’s responses was not feasible. Therefore, the primary focus of the research is on the overall use of invitations during the substantive phase of the interviews and age differences in children’s elaboration in response to those invitations.

#### Inter-Rater Reliability

The transcripts were manually coded by native speakers who were blind to the study’s hypothesis and purpose. The raters underwent training in the coding procedure by an experienced coder (the first author) using an independent set of interview transcripts. This training continued until the raters achieved 90% agreement on the classification of the interviewer’s question types and children’s responses. To ensure ongoing reliability, 30% (*n* = 76) of the transcripts were independently coded by both raters. The remaining interviews were evenly divided between the two coders. The coding demonstrated excellent inter-rater reliability, with Cohen’s κ = .89 for interviewers’ prompts and Cohen’s κ = .91 for children’s responses.

### Statistical Analysis

All analyses were conducted using SPSS version 28 and Stata. Descriptive statistics were calculated to examine the frequency of interviewers’ use of main and cued invitations during the substantive phase of the interviews, the distribution of the first main versus cued invitations posed across the interviews, and children’s elaboration in response to main versus cued invitations. Non-parametric tests were conducted to analyze differences in the distribution of the first main invitations versus cued invitations across the interviews. Logistic regression analyses with standard errors adjusted for intrasubject correlation, to account for repeated measurements, were conducted to examine differences in children’s elaboration of event-specific, forensically relevant information in response to main versus cued invitations, children’s age groups (2–4 years vs. 5–6 years), and children’s elaboration of forensically relevant information related to the investigated incident(s) and the timing of invitations posed during the interviews.

## Results

In the substantive part of the interviews, investigators addressed an average of 154,23 substantive prompts that explicitly addressed information and details about the investigated incident. Among these substantive prompts, a total of 1065 were classified as invitations (main invitations and cued invitations collapsed), representing 2.7% of the total amount of substantive prompts in the sample (*n* = 39,483). Of these invitations, forty-three (4.0%) were classified as *main invitations*, while 1022 (95.7%) were classified as *cued invitations*.

### Frequency of Main Invitations

Main invitations (*n* = 43) were posed in 32 (12.5%) of the 256 interviews. The number of main invitations posed in these interviews ranged from one to two (*M* = 1.34, *SD* = 0.48) with one invitation posed in 21 interviews, and two invitations posed in 11 interviews. In the remaining 224 interviews (87.5%), main invitations were not used to elicit or facilitate information regarding the investigated incident. The first main invitation was introduced, on average, in turn 122.68 (*SD* = 88.18; range: 1–321) in the substantive phase of the interviews. Most of the main invitations (*n* = 33) were posed in interviews with children in the upper age group (5–6 years, *n* = 23), compared to the lower age groups (2–4 years, *n* = 9).

### Frequency of Cued Invitations

Cued invitations (*n* = 1022) were posed in 229 (89.5%) of the 256 interviews. On average, 4.4 cued invitations (*SD* = 5.42) were posed across these interviews, with a considerable range from 1 to 55. The first cued invitation was introduced, on average, in turn 75.38 (*SD* = 74.88; range: 1–399) in the substantive phase of the interviews.

### Distribution of the First Main Invitations and Cued Invitations Posed Across the Interviews

**Figure 1.**
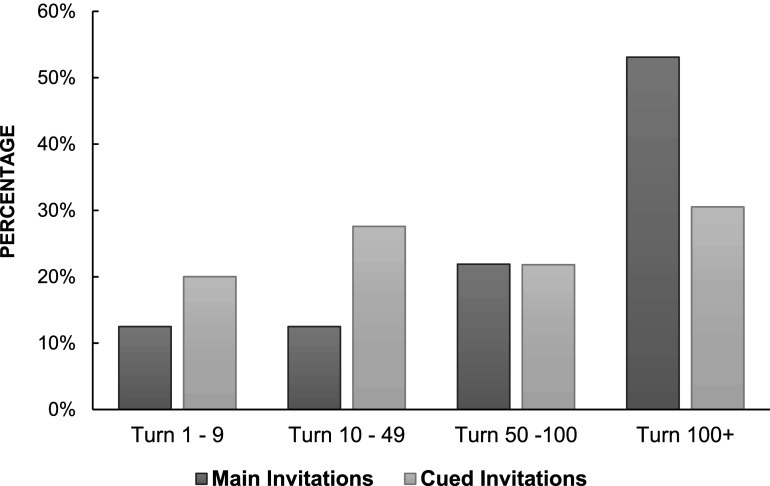
Distribution of the first main versus cued invitation posed across the interviews.

Figure 1 visualizes the differences in the distribution of the first main and cued invitations posed during the substantive phase of the interviews. A Mann-Whitney U test was conducted to determine whether there was a significant difference in the distribution of the first main invitations compared to the first cued invitations in the substantive phase of the interviews. The results revealed a statistically significant difference, indicating that main invitations were posed later in the substantive phase than cued invitations, *U* = 31,584, *p* < .001.

### Children’s Response Patterns to Main Invitations Versus Cued Invitations

**Figure 2.**
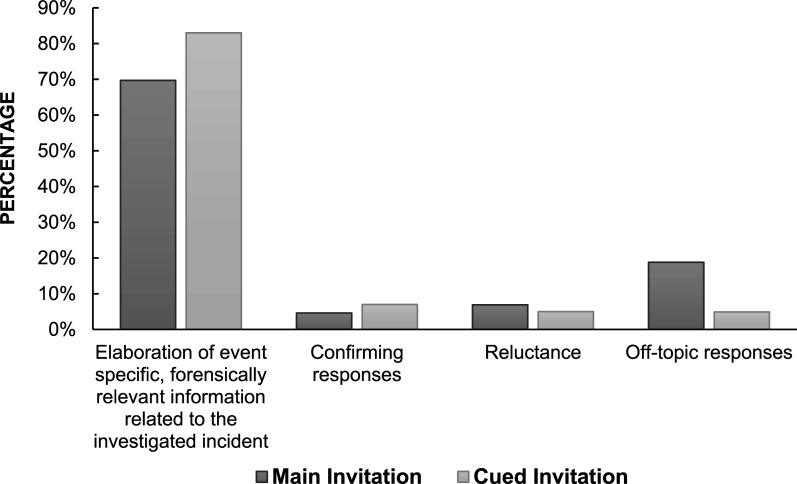
Children’s elaboration in response to main versus cued invitations. *Note*. Denial (0.8%) and correcting responses (0.3%), which where only present in response to cued invitations, were not included in the figure.

Figure 2 illustrates children’s elaboration in response to main versus cued invitations, regardless of age. The predominant response for both invitation types was the provision of event-specific, forensically relevant information related to the investigated incident. Specifically, 69.7% of responses to main invitations elicited elaboration of event specific, forensically relevant information from the children. A slightly higher percentage (83%) of responses to cued invitations elicited similar information. Additionally, 4.6% of children’s responses to main invitations consisted of confirming responses, which did not provide additional forensically relevant information. This resulted in a total elaboration rate of 74.3% for main invitations and 90% for cued invitations. A Chi-Square test of independence was conducted to assess differences in the frequency of elaboration and confirming responses elicited by main versus cued invitations. The analysis revealed no statistically significant difference in response types between the two groups, χ^2^(2, *N* = 272) = 3.76, *p* = .152. Although cued invitations elicited a higher proportion of event-specific responses, the odds of eliciting forensically relevant information in response to main invitations vs. cued invitations were not statistically significant. A logistic regression analysis was conducted to examine the potential differences in children’s elaboration of event-specific, forensically relevant information when interviewers posed main versus cued invitations. The odds ratio for elaboration in response to main versus cued invitations was not statistically significant [OR] = 2.3, 95% confidence interval [CI] [0.85, 6.23]).

### Children’s Elaboration of Forensically Relevant Information and Age

A logistic regression analysis was performed to examine the association between age groups (2–4 years vs. 5–6 years) and provision of forensically relevant information related to the investigated incident(s). Children aged 5–6 years exhibited significantly higher elaboration in response to both main and cued invitations compared to children aged 2–4 years (OR = 2.60, 95% CI: 1.06, 6.37, *p* = .036).

### Timing of Main Versus Cued Invitations

A logistic regression analysis was conducted to examine the association between children’s elaboration of forensically relevant information and the timing of invitations posed during the interviews, measured in turns (question/response pairings). The results revealed a significant association, with earlier linked to higher elaboration (OR = 0.99, 95% CI: 0.996, 0.999, *p* = .001). Specifically, for every additional unit increase in turn, the odds of elaboration decreased by approximately 0.3%.

### Interviewers’ Follow-Up Prompts Following Main Invitations and Cued Invitations

**Figure 3.**
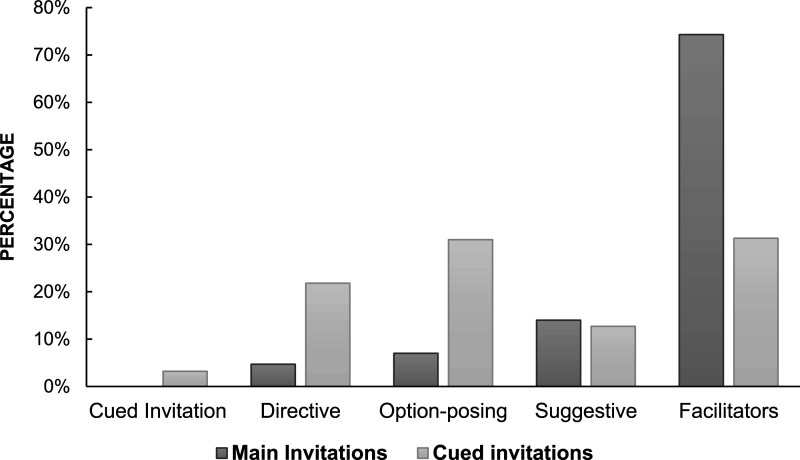
Distribution of follow-up prompts to children’s responses to main and cued invitations.

Figure 3 illustrates the distribution of interviewer’s follow-up prompts after the children’s elaboration of forensically relevant information in response to main and cued invitations. Facilitators were the most frequently employed follow-up prompt after both types of invitations. Notably, for cued invitations, facilitators and option-posing prompts were used in equal frequency. In 3.2% (*n* = 33) of the interviews, the first cued invitation was followed by another cued invitation, whereas none of the main invitations were followed by another main invitation or cued invitation. The distribution of suggestive questions was nearly identical for both types of invitations, comprising 12.7% of the follow-up questions asked after cued invitations and 14% of the follow-up questions asked after main invitations.

### Children’s Elaboration in Response to Follow-Up Prompts

When interviewers posed a subsequent cued invitation after the initial one, children continued to provide additional forensically relevant information in 93.4% (*n* = 31) of cases. A majority of children also continued to elaborate on forensically relevant details when follow-up prompts were posed as directive questions (69.7%, *n* = 154) or facilitator utterances (68.1%, *n* = 220). In contrast, the lowest rates of additional elaboration were observed following option-posing (26.1%, *n* = 65) and suggestive (28.7%, *n* = 37) follow-up prompts. A chi-square test of independence was performed to examine the relationship between the types of follow-up questions and children’s elaboration of subsequent forensically relevant information. The analysis revealed a statistically significant association between these variables, *X*^2^(4, *N* = 1022) = 234.9, *p* = .001), indicating that the type of follow-up question is related to the likelihood of eliciting further forensically relevant information from the children.

## Discussion

The present investigation revealed distinct patterns in interviewers’ use of main and cued invitations, as well as children’s response behaviors, in a national sample of 256 investigative interviews with preschool-aged children. Across these interviews, 1065 invitations were posed that directly addressed the investigated incident. Notably, the vast majority (95.7%, *n* = 1022) were classified as *cued invitations*, which used details previously disclosed by the child to formulate open-ended follow-up prompts. In almost 90% of the interviews, at least one cued invitation was employed to facilitate the child’s recollection of the incident, indicating that interviewers are somewhat familiar with using cued invitations in forensic interviews. However, the average of 4.4 cued invitations per interview is significantly lower than expected based on best practices. While these invitations are crucial for prompting children to elaborate on prior disclosures and for eliciting richer narratives, their lower-than-expected frequency suggests that interviewers may not be fully capitalizing on their potential. Nonetheless, the discrepancy between recommended guidelines and actual practice could reflect limited adherence to best practices or a lack of confidence in using invitations. Given the integral role of cued invitations in obtaining forensically relevant information while minimizing suggestiveness, these results underscore a significant gap between current practice and recommended guidelines. In contrast, main invitations-which allow the interviewees to freely recall their accounts without being direct prompting- were posed far less frequently. They comprised 4% (*n* = 43) of all invitations across the interviews and were used in 12% of cases, meaning that 87% of interviews lacked any main invitations during the substantive phase of the interviews. Several factors may contribute to the predominant use of cued invitations over main invitations when facilitating preschool-aged children’s recollections of abuse. One potential factor is the concept of self-fulfilling expectations. Interviewers may notice that children tend to elaborate more readily in response to cued invitations, reinforcing a preference for these types of questions. Additionally, concerns about young children tiring quickly might prompt interviewers to a more direct approach to maximize efficiency. This reliance on using fewer main invitations can be viewed as a strategy to “cut to the chase”, allowing interviewers to gather the desired information more swiftly. As children continue to respond positively to cued invitations, this success may further entrench the practice, creating a cycle in which interviewers become increasingly hesitant to employ main invitations. Consistent findings regarding the underuse of main invitations have led scholars to explore the barriers to their use, identifying several practitioner-based assumptions that may limit reliance on such prompts (Guadagno et al., 2006, 2013; Wright & Powell, 2006; Yii et al., 2014). One possible explanation for the preference for cued invitations is that interviewers rely on them due to their greater familiarity and perceived efficacy. Moreover, many practitioners view main invitations as ineffective for eliciting specific details needed for investigative purposes. For instance, Guadagno et al. (2006) found that practitioners often dismissed main invitations, opting instead for more specific questions to obtain crucial investigative details such as the identity of the alleged offender, their intent and motives, and the timing and occurrence of the abuse. Moreover, Wright & Powell (2006) examined the challenges trained interviewers face in maintaining an open-ended questioning style and identified a primary barrier; the need to gather specific information to verify allegation, establish corroborating evidence, and support legal charges. Consequently, interviewers often default to more specific questions-even though open-ended prompts are known to elicit more detailed and reliable accounts from children. Additionally, Wright & Powell (2006) noted that the unfamiliarity with open-ended discourse styles and the difficulty in distinguishing between open-ended and specific further contribute to the reluctance to adopt an open-ended approach. Moreover, the underuse of open-ended prompts may reflect a lack of understanding about their value, as research have shown that interviewers’ knowledge of such prompts is linked to their adherence to best practices (Yii et al., 2014). Collectively, these findings suggest that despite substantial evidence supporting the effectiveness of invitations, practitioners’ perception of their ineffectiveness may lead to their dismissal in practice.

The main findings in the current study, along with previous research, challenge the belief that main invitations are ineffective in eliciting forensically relevant information from young children. In fact, evidence suggests that main invitations can effectively elicit forensically relevant details for prosecution purposes. Even without specific cues, children as young as 3 years old can provide valuable forensic information in response to these open-ended prompts (e.g., Cyr & Lamb, 2009; Danby & Sharman, 2023; Gagnon & Cyr, 2017; Hershkowitz, 2001; Lamb et al., 2003, 2007b; Orbach & Lamb, 2000). Our findings further indicate that both main and cued invitations were equally effective in eliciting event-specific, forensically relevant information from the children. This finding suggests that preschool-aged children may have a greater capacity for detailed recall than previously assumed, regardless of whether they are responding to main or cued invitations. Specifically, nearly 70% of responses to main invitations and 83% of responses to cued invitations provided event-specific information that enhanced the understanding of the investigated incidents. This information included details about the events, objects, and individuals involved. Notably, children aged 5–6 years were more likely to offer forensically relevant details compared to those aged 2–4 years. These results contradict the common assumption that preschoolers struggle to provide informative responses to invitations and challenge the belief that focused prompts are necessary to elicit event-specific details from young children. Instead, our findings-consistent with previous research (Lamb et al., 2003, 2007b) support the recommendation that interviewers should develop and prioritize the skill of using main invitations before turning to more focused questions allowing the children the opportunity to freely recall their memories. Only afterward should more focused, cued questions be employed, thereby minimizing reliance on recognition-based responses (Lamb et al., 2003, 2007b).

In addition to the infrequent use of invitations, our study revealed notable patterns regarding the *timing* of these prompts aimed at eliciting children’s recollection of the target incident. Contrary to best practice recommendations-which advocate for introducing invitations early when addressing questions about the investigated incident-main invitations were often posed late in the interviews. Notably, the first main invitations were introduced, on average, around turn 122 in the substantive phase, significantly later than the first cued invitations, which occurred at an average of turn 75. In majority of interviews (75%), the first invitation was posed after turn 50, and in over half (52%) of the interviews the first cued invitation was posed after turn 50. These findings suggest that neither main nor cued invitations are being used systematically or in a structured manner to tap into children’s free-recall memory early in the substantive phase. Instead, invitations tend to be used sporadically and later in the interview, after children have already been subjected to a considerable number of other questions about the incident. Another key finding was that interviewers typically present only one invitation before switching to other question types. Specifically, after the initial invitation, interviewers predominantly employed directive and option-posing prompts, along with a notable proportion of suggestive prompts. Only 3% of interviews included additional invitations in subsequent turns. Similar studies have reported comparable findings, showing that even when children provide forensically relevant information in response to open-ended prompts, subsequent questions are often closed-ended (Korkman et al., 2008; Wolfman et al., 2016). Moreover, differences in the patterns of interviewers’ follow-up questions were also evident. Cued questions were frequently followed by closed questions, while the rare main invitations tended to be followed more often by facilitators. This difference can be attributed to several factors related to interview dynamics. Cued invitations are typically more focused and direct, prompting specific information from the child. Interviewers may find that following these with closed questions is an efficient way to elicit precise details or clarify specific points without deviating from the topic. Furthermore, when children elaborate in response to cued invitations, it may signal that they are engaged and capable of providing further detailed information, prompting interviewers to use closed questions to confirm these specific details. Importantly, follow-up invitations proved to be more effective than all other question types in consistently eliciting forensically relevant information from children in subsequent turns. When interviewers posed a subsequent cued invitation after the initial one, children provided additional forensically relevant information in 93% of cases. This finding suggests that follow-up invitations can be as effective as main invitations in eliciting further forensically relevant information from children.

Our study also uncovered another significant pattern concerning the effectiveness of invitations over time. As the interviews progressed, invitations posed later were notably less effective at eliciting productive responses compared to those used earlier. This finding suggests that the capacity of invitations to draw out forensically relevant information from preschoolers can be more potent when employed at the beginning of the interview. These findings underscore best practice recommendations for conducting investigative interviews with children, emphasizing the critical importance of strategically incorporating invitations early in the interview to ensure that interviewees are invited to freely recall events in their own words (Korkman et al., 2024; Lamb et al., 2007a, 2008). Recognizing this diminishing effectiveness of invitations as an interview unfold emphasizes the need for interviewers to employ these questioning techniques at the outset.

In summary, this study demonstrates that invitations can effectively elicit forensically relevant information from children as young as 3 years old, aligning with a growing body of evidence (Gagnon & Cyr, 2017; Lamb et al., 2008). Despite children’s ability to provide detailed information in response to invitations, many interviewers struggled to adhere to best practice recommendations, which recommend inviting interviewees to freely recall their accounts. Instead, interviewers frequently rely on focused questions when facilitating young children’s recollection of abuse. Alongside the low number of invitations, interviews often discontinue invitations after the initial one, limiting the opportunities to gather additional forensically relevant information. These patterns may indicate that, as reported by practitioners (Guadagno et al., 2006, 2013; Wright & Powell, 2006), interviewers remain unconvinced of the benefits of invitations in eliciting information from young children.

### Implications for Practice of Investigative Interviews and Directions for Future Research

The recurring patterns observed across multiple studies indicate that initiating main invitations, *timing* them effectively, and *maintaining* their use beyond the initial prompt are challenging for interviewers. Findings from real-world investigative interviews including those in the current study- emphasize the necessity of specialized training programs aimed at systematically developing these skills to maximize the elicitation of productive responses from children (Baugerud et al., 2024; Korkman et al., 2024; Powell, 2013; Røed et al., 2023; Talwar et al., 2024). Given the observed decline in the effectiveness of invitations over time, the timing of invitations is particularly critical. Training should ensure that interviewers understand the distinctions among various question types, appreciate the advantages of open-ended prompts, and become familiar with the different subtypes (Yii et al., 2014). Mastering open-ended questioning is a complex skill that requires an in-depth understanding of its nuances (Powell, 2013). Innovative training programs that incorporate virtual child avatars offer unique advantages by providing a realistic yet controlled environment that simulates various scenarios encountered in real-life interviews (Baugerud et al., 2024). Research integrating avatars into training has demonstrated that simulated practice is a promising avenue for enhancing proficiency in using invitations effectively (e.g., Baugerud et al., 2024; Benson & Powell, 2015; Brubacher et al., 2025; Haginoya et al., 2025; Hassan et al., 2023; Kask et al., 2022; Pompedda et al., 2015; Powell, 2013; Røed et al., 2023; Salehi et al., 2022). Therefore, specialized, evidence-based training programs tailored for interviews with preschool-aged, alleged victims should strategically prioritize development of skills in using various subtypes of invitations. Additionally, gaining a more in-depth understanding of the factors that facilitate or limit adherence to best practice recommendations—particularly those encourage the early introduction of invitations—requires a thorough exploration of practitioners’ experiences. This approach, as demonstrated in the field (e.g., Guadagno et al., 2006, 2013; Wright & Powell, 2006; Yi et al., 2014), would provide the most effective means of addressing these barriers.

### Limitations

The present study has several limitations. First, our findings are specific to investigative interviews conducted by police investigators during criminal investigations and may not be applicable to other interview settings, such as interviews carried out by child protective services. Research has shown significant differences in interview techniques across these contexts (Baugerud et al., 2023), highlighting the importance of considering contextual factors. Although the study sample is large, the relatively small number of interviews within each age group presents a potential limitation. This limited age-specific data may reduce the ability to identify age-related trends or draw robust conclusions about differences between age groups. Future research should consider larger subgroups to address this issue more effectively. Additionally, our exploration of the effects of invitation subtypes on preschool children’s productivity was based solely on the children’s response patterns during the interviews. We were unable to assess the accuracy of the information obtained through main and cued invitations in the interviews.

Another potential limitation of this study is the non-independence of responses due to multiple questions being derived from a single interview, even though we accounted for intrasubject correlation using standard errors adjusted for repeated measurements in the statistical analyses. Lastly, our investigation into age-related differences in responses to invitations focused on two broad age groups (2- to 4-year-olds and 5- to 6-year-olds). While this division provided a preliminary understanding of age-related trends, a more detailed examination using narrower age ranges could offer a more comprehensive understanding of developmental differences in responsiveness to invitations.

### Conclusions

This study indicates that children as young as 3 years old can provide forensically relevant information through both main and cued invitations in a real-life investigative interview. Notably, both invitation types were equally effective, challenging the prevailing assumption that young children require additional cues to provide forensically relevant information in formal investigative interviews. The findings also revealed a significant decline in the effectiveness of invitations as interviews progress, emphasizing the heightened impact of administering them early in the process. These results emphasize the need for targeted training programs that systematically develop interviewers’ skills in initiating, timing, and maintaining the use of invitations to maximize the retrieval of accurate and detailed information from young children.
